# The Role of the Na^+^/Ca^2+^ Exchanger in Aberrant Intracellular Ca^2+^ in Cardiomyocytes of Chagas-Infected Rodents

**DOI:** 10.3389/fcimb.2022.890709

**Published:** 2022-07-07

**Authors:** Jose R. Lopez, Nancy Linares, Jose A. Adams, Alfredo Mijares

**Affiliations:** ^1^ Department of Research, Mount Sinai, Medical Center, Miami, FL, United States; ^2^ Centro de Biofísica y Bioquímica, Instituto Venezolano de Investigaciones Científicas, Caracas, Venezuela; ^3^ Division of Neonatology, Mount Sinai, Medical Center, Miami, FL, United States

**Keywords:** chagas disease, Na/Ca exchanger, cardiomyopathy, *Trypanosoma cruzi* (T cruzi), calcium

## Abstract

Chagas disease is produced by the parasite *Trypanosoma cruzi (T. cruzi)*, which is the leading cause of death and morbidity in Latin America. We have shown that in patients with Chagas cardiomyopathy, there is a chronic elevation of diastolic Ca^2+^ concentration ([Ca^2+^]_d_), associated with deterioration to further address this issue, we explored the role Na^+^/Ca^2+^ exchanger (NCX). Experiments were carried out in noninfected C57BL/6 mice and infected with blood-derived trypomastigotes of the *T. cruzi* Y strain. Anesthetized mice were sacrificed and the cardiomyocytes were enzymatically dissociated. Diastolic [Ca^2+^] ([Ca^2+^]_d_) was measured using Ca^2+^ selective microelectrodes in cardiomyocytes from control mice (CONT) and cardiomyocytes from *T. cruzi* infected mice in the early acute phase (EAP) at 20 dpi, in the acute phase (AP) at 40 dpi, and in the chronic phase (CP) at 120 dpi. [Ca^2+^]_d_ was 1.5-times higher in EAP, 2.6-times in AP, and 3.4-times in CP compared to CONT. Exploring the reverse mode activity of NCX, we replaced extracellular Na^+^ in equivalent amounts with N-methyl-D-glucamine. Reduction of [Na^+^]_e_ to 65 mM caused an increase in [Ca^2+^]_d_ of 1.7 times in cardiomyocytes from CONT mice, 2 times in EAP infected mice, 2.4 times in AP infected mice and 2.8 in CP infected mice. The Na^+^ free solution caused a further elevation of [Ca^2+^]_d_ of 2.5 times in cardiomyocytes from CONT, 2.8 times in EAP infected mice, 3.1 times in AP infected mice, and 3.3 times in CP infected mice. Extracellular Ca^2+^ withdrawal reduced [Ca^2+^]_d_ in both CONT and cardiomyocytes from Chagas-infected mice and prevented the increase in [Ca^2+^]_d_ induced by Na^+^ depletion. Preincubation with 10µM KB-R7943 or in 1µM YM-244769 reduced [Ca^2+^]_d_ in cardiomyocytes from infected mice, but not control mice. Furthermore, both NCX blockers prevented the increase in [Ca^2+^]_d_ associated with exposure to a solution without Na^+^. These results suggest that Ca^2+^ entry through the reverse NCX mode plays a significant role in the observed [Ca^2+^]_d_ dyshomeostasis in Chagas infected cardiomyocytes. Additionally, NCX inhibitors may be a viable therapeutic approach for treating patients with Chagas cardiomyopathy.

## Introduction

Chagas disease, caused by the protozoan *Trypanosoma cruzi* (*T. cruzi*), affects approximately eight million people in the poorest areas of Central and South America. The prevalent form of transmission of *T. cruzi* to humans is contact with contaminated feces/urine of the invertebrate vector during a blood meal. However, human infection through blood transfusion, organ transplantation and oral. (Bern et al., 2008) and oral transmission due to ingestion of contaminated fruit juices (Alarcon De Noya et al., 2010) have been reported. Chagas disease is characterized by a varied clinical scenario that evolves from an acute phase, often asymptomatic or oligosymptomatic, to a chronic phase that can manifest as indeterminate, cardiac, or digestive forms (Echavarria et al., 2021). Most infected people develop cardiac conditions characterized by ventricular arrhythmias, heart blocks, heart failure, thromboembolic phenomena, and sudden death (Rassi et al., 2010; Teixeira et al., 2011; Echavarria et al., 2021; http://www.who.int/mediacentre/factsheets/fs340/en/, 2021).

Calcium is a central player in the regulation of cardiac contractility, and its intracellular concentration is maintained through the interaction of Ca^2+^ transport mechanisms and diastolic leak mediated by the ryanodine receptor (Shannon et al., 2003; Bers and Ginsburg, 2007; Bers, 2014; Freichel et al., 2017; Smith and Eisner, 2019). In healthy cardiomyocytes, the diastolic Ca^2+^ concentration ([Ca^2+^]_d_) is in the range of 100-120 nM (Marban et al., 1980; Lopez et al., 2011; Mijares et al., 2014; Mijares et al., 2020). We have demonstrated dysregulation of [Ca^2+^]_d_ in cardiomyocytes isolated from Chagas patients, which is correlated with the patient’s clinical condition (Lopez et al., 2011). Furthermore, this diastolic disturbance of [Ca^2+^] appears to be associated with alterations in the regulation of the intracellular messenger inositol 1,4,5-trisphosphate (Lopez et al., 2011; Mijares et al., 2020).

NCX is a bidirectional high-capacity transporter that represents the primary way of Ca^2+^ extrusion in excitable cells (Blaustein and Lederer, 1999; Aronsen et al., 2013). It can import 3 Na^+^ into the cell in exchange for 1 Ca^2+^ (forward mode) or drive the efflux of 3 Na^+^ in exchange for 1 Ca^2+^ (reverse mode) across the plasma membrane (Bers and Ginsburg, 2007). The direction of NCX (forward or reverse) depends on the internal and external concentration of both Na^+^ and Ca^2+^ and the membrane potential (Bers, 2000). NCX works almost exclusively in the forward mode (Ca^2+^ extrusion) in normal cardiomyocytes, driven primarily by the elevated subsarcolemmal [Ca^2+^]. Elevated diastolic [Na^+^] ([Na^+^]_d_) could change the direction of the fluxes to a more significant influx of Ca^2 +^ and fewer Ca^2 +^ effluxes, resulting in increased intracellular [Ca^2+^]. However, the dynamic of NCX can be altered in pathophysiological situations such as heart failure, arrhythmia, ischemia-reperfusion injury, and hypertrophy (Bers and Despa, 2006). Recently, Santos-Miranda et al. (Santos-Miranda et al., 2021) reported a cellular arrhythmogenic profile in chronic Chagas cardiomyopathy, which was sensitive to Ni^2+^ and NCX blocker SEA0400, indicating the involvement of NCX.

In the present study, we evaluated the activity of NCX in cardiomyocytes from control and Chagas infected mice. We showed that NCX activity is enhanced in cardiomyocytes from *T. cruzi-*infected mice compared to control cardiomyocytes and that the reverse mode of NCX contributes to intracellular Ca^2 +^ dyshomeostasis observed in cardiomyocytes from infected mice.

## Material and Methods

### Animals

Homozygous male C57BL/10 with a bodyweight of 25 ± 0.45 g. and 12 weeks of age were housed at constant temperature (24 C) and a 12 h light/12 h dark cycle, with free access to water and fed a commercial pelletized diet.

### *Trypanosoma cruzi* Infection

Mice were randomly divided into two groups: **Group A** (Control) that were not inoculated with *T. cruzi* and **Group B** (infected mice) were infected intraperitoneally with 5x10^-2^ blood-form trypomastigotes of the Y strain of *T. cruzi* (O’Daly et al., 1984). The Y strain of *T. cruzi* was originally obtained from patients in Brazil and was kindly provided by Dr. Jose O’Daly, who has maintained the strain over the years through serial passages in homozygous C57B1/6 mice. Parasitemia was monitored by counting the number of parasites/5 μl of blood obtained from the tail vein of infected mice. A positive parasitemia was found in all inoculated animals on days 12-15 post-infection (dpi). Non-infected and infected anesthetized (ketamine/xylazine) mice were sacrificed by cervical dislocation at 20 (early acute phase), 40 (acute phase), and 120-dpi (chronic phase), and the cardiomyocytes were enzymatically dissociated.

### Isolation of Cardiomyocytes and Inclusion Criteria

Control and infected mice were anesthetized (ketamine 100 mg/kg/xylazine 5 mg/Kg) and hearts were rapidly removed, attached to a cannula, and mounted on a Langendorff reverse coronary perfusion system for the enzymatic dissociation of ventricular cardiomyocytes (Liao and Jain, 2007). Only Ca^2+^ tolerant cardiomyocytes with rod-shaped, well-defined striation spacing and a resting sarcomere length of ≥1.75 µm (Wussling et al., 1987) were used and studied within 4-6 h after isolation. The isolation procedure was carried out at 37°C.

### Ca^2+^ and Na^+^ Selective Microelectrodes

Double-barreled Ca^2+^ and Na^+^ selective microelectrodes were prepared as previously described (Eltit et al., 2013). Each ion-selective microelectrode was individually calibrated before and after the determination of [Ca^2+^]_d_ and the [Na^+^]_d_ as previously described (Mijares et al., 2014). Ca^2+^ selective microelectrodes with a Nernstian response between pCa3 and pCa7 (30.5 mV/pCa units at 37°C) and Na^+^ selective microelectrodes with Nernstian responses between 100 and 10 mM [Na^+^]_e_, and an adequate response (40–45 mV) between 10 and 1 mM [Na^+^]_e_ were used experimentally (Lopez et al., 1983; Lopez et al., 2000; Eltit et al., 2013). The Ca^2+^- and Na^+^-microelectrode response was not affected by any of the drugs used in the present study.

### Recording of Diastolic Calcium and Sodium Concentrations

Isolated cardiomyocytes from control mice and *T. cruzi* infected mice were impaled with double-barrel Ca^2+^ or Na^+^ microelectrodes to measure [Ca^2+^]_d_ and [Na^+^]_d_, respectively, and potentials were recorded using a high impedance electrometer (WPI 773 electrometer, FL, USA) as previously described (Lopez et al., 2000; Lopez et al., 2017). The potential of the 3M KCl microelectrode barrel (Vm) was electronically subtracted from the potential recorded by the Ca^2+^ selective barrel (V_Ca_) to produce a differential Ca^2+^ specific potential (V_CaE_) that represents the cardiomyocyte [Ca^2+^]_d_. A similar approach was used to produce the specific Na^+^ potential (V_NaE_), which represents the cardiomyocyte [Na^+^]_d_. The potentials were acquired at a frequency of 1,000 Hz with the AxoGraph software (version 4.6; Axon Instruments, CA, USA) and stored in a computer for further analysis.

Individual cardiomyocyte measurements of [Ca^2+^]_d_ and [Na^+^]_d_ from control and infected mice were accepted when there was ***i)*** an abrupt drop to a steady level of Vm equal to or more negative than -80 mV in healthy cells and -70 mV in unhealthy cardiomyocytes; ***ii)*** a stable recording of Vm and V_Ca_ potentials or Vm and V_Na_ potentials for more than 60 seconds and an abrupt return to baseline at the exit of the microelectrode from the cell. Vm was used as a biological indicator of the integrity of cardiomyocyte sarcolemma in real-time; therefore, any cardiomyocytes with a recorded Vm <-70 mV were discarded. These criteria were not met in 18% of total impalements performed in cardiomyocytes from control mice and 41% in infected mouse cardiomyocytes.

### Reverse Mode NCX Protocol

The effect of NCX (reverse mode) on [Ca^2+^]_d_ in cardiomyocytes from control and infected mice was determined by preincubating cardiomyocytes in a solution in which [Na^+^]e was partially or completely removed (see solutions below) and replaced by *N*-methyl-d-glucamine hydrochloride. Measurements of [Ca^2+^]_d_ were carried out before and after incubation in low Na^+^ solutions.

### Equilibrium Potentials

The equilibrium potentials for Ca^2+^ (*E*_Ca_) and Na^+^ (*E*_Na_) were calculated using the Nernst equation (*E_ion_=* RT/zF x ln [ion]_i/_[ion]_e_) and [Ca^2+^]_d_ and [Na^+^]_d_ obtained in cardiomyocytes from CONT and infected mice in EAP, AP, and CP. The equilibrium potential of the Na^+^/Ca^2+^ exchanger (*E*_NCX_) was estimated using the following equation: *E_NCX_* = 3E_Na_ - 2E_Ca_ (with a stoichiometry of 3:1), where *E*_Na_ and *E*_Ca_ are the equilibrium potentials of Na^+^ and Ca^2+^ obtained from the Nernst equation.

### Solutions

All solutions were made with ultrapure water supplied by a Milli-Q system (Millipore, Bedford, MA). The Tyrode solution had the following composition (in mM): 130 NaCl, 5 KCl, 2.5 CaCl_2_, 1 MgCl_2_, 20 NaHCO_3_, 0.33 NaH_2_PO_4_, and 10 glucose gassed with 95% O_2_ and 5% CO_2_, pH 7.4. A low- or Na^+^-free solution was prepared by the partial or total withdrawal of [Na^+^]_e_, and replacement by an equivalent amount of impermeable cation *N*-methyl-d-glucamine (NMG) hydrochloride to maintain osmolarity. A Ca^2 +^-free solution was prepared, omitting CaCl_2_ and adding 1 mm of EGTA and 2 mm of MgCl_2_. Solutions 2-(2-(4-(4-nitrobenzyloxy-phenyl-ethyl-isothiourea methanesulfonate (KB-R7943) and N- (3-aminobenzyl) -6- 4- [(3-fluorobenzyl-oxy] phenoxy nicotinamide (YM-244769) were made by adding the desired concentration of the reagent to the Tyrode solution. All experiments were carried out at 37°C.

### Statistical Analysis

Experimental results are expressed as means ± SD; *n_mice_* represents the number of mice used and *n_cells_* the number of successful measurements in cardiomyocytes isolated from CONT and infected mice used for statistical analysis. Data were analyzed using one-way analysis of variance ANOVA for repeated measures, followed by Tukey’s multiple comparison tests to determine significance. A *p*<0.05 was considered significant. GraphPad Prism 9 (GraphPad Software, CA, USA) was used for statistical analysis.

## Results

### [Ca^2+^]_d_ and [Na^+^]_d_ in Ventricular Cardiomyocytes of Chagasic Rodents

**Figure 1:**
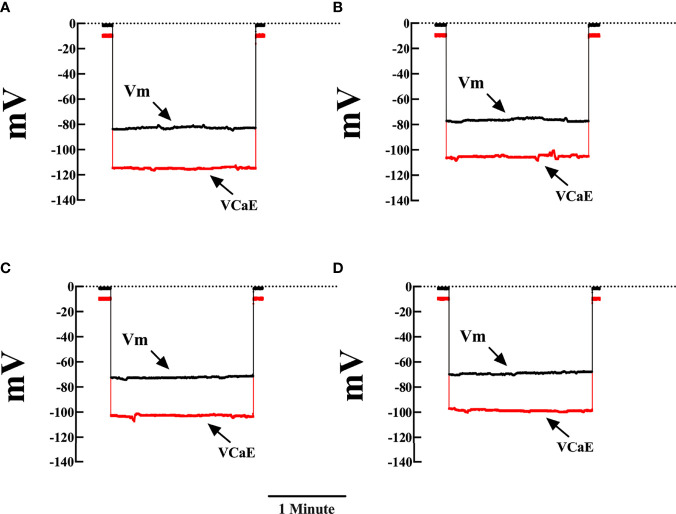
Representative measurements of Vm and [Ca^2+^]_d_ in cardiomyocytes from control and Chagas infected mice. Simultaneous measurements of membrane potential (Vm) and [Ca^2+^]_d_ **(A)** Cardiomyocyte from control mice (Vm was -82 mV and [Ca^2+^]_d_ 122 nM). **(B)** Cardiomyocyte from EAP-infected mice (Vm was -76mV and [Ca^2+^]_d_ 200 nM). **(C)** Cardiomyocyte from AP-infected mice (Vm was -73 mV and [Ca^2+^]_d_ 312 nM). **(D)** Cardiomyocyte from CP-infected mice (Vm was -69 mV and [Ca^2+^]_d_ 414 nM). Vm, resting membrane potential and V_Cae_, specific calcium potential; CONT, control mice; EAP, infected mice in the early acute phase; AP, infected mice in the acute phase; and CP, infected mice in the chronic phase. Calibration bar = 1 minute.

**Figure 2:**
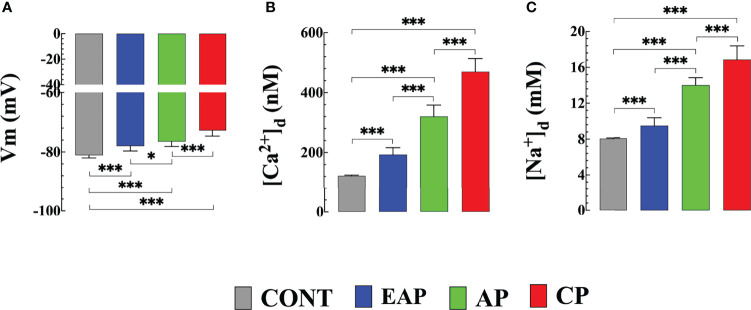
Resting membrane potential, diastolic [Ca^2+^] and [Na^+^] in cardiomyocytes from control and Chagas infected mice. **(A)** The resting membrane potential, **(B)** [Ca^2+^]_d,_ and **(C)** [Na^+^]_d_ were measured in quiescent cardiomyocytes from CONT mice (*n_cells_* =24 and *n_mice_* =3), EAP-infected mice (*n_cells_* =19 and *n_mice_* =3), AP-infected mice (*n_cells_* =21 and *n_mice_* =3) and CP-infected mice (*n_cells_* =18 and *n_mice_* =5). Data are expressed as means ± S.D. Statistical analysis was performed using one-way ANOVA, followed by Tukey’s multiple comparison tests, *p < 0.05; ***p < 0.001. CONT, control mice; EAP, early acute phase of infected mice; AP, acute phase of infected mice; and CP, chronic phase of infected mice.

We previously reported a significant elevation in [Ca^2+^]_d_ in cardiomyocytes obtained from patients with chronic Chagas cardiomyopath, which was related to the extent of their cardiac dysfunction (Lopez et al., 2011; Mijares et al., 2020). **Figure 1** shows representative records of simultaneous measurement of Vm and [Ca^2+^]_d_ in a single cardiomyocyte isolated from (**A**) CONT mice, (**B**) EAP-infected mice (20 dpi), (**C**) AP-infected mice (40 dpi), and (**D**) CP-infected mice (120 dpi). The cardiomyocytes of Chagas-infected mice showed partial depolarization and increased [Ca^2+^]_d_, which aggravated as a function of days after infection. The mean Vm and [Ca^2+^]_d_ in quiescent cardiomyocytes from CONT mice were 81 ± 1 mV and 122 ± 3 nM. Respectively. In cardiomyocytes from EAP-infected mice, Vm was 78 ± 1.7 mV and [Ca^2+^]_d_ 194 ± 23 nM (*p*<0.001 compared to CONT cardiomyocytes); in cardiomyocytes from AP-infected mice, Vm was 76 ± 1.6 mV and [Ca^2+^]_d_ 320 ± 38 nM (*p*<0.001 compared to CONT cardiomyocytes) and in cardiomyocytes from CP-infected mice, Vm was 72 ± 1.8 mV and [Ca^2+^]_d_ 470 ± 43 nM (*p*<0.001 compared to CONT cardiomyocytes) (**Figures 2A, B**).

Similar differences in [Na^+^]_d_ were obtained in the measurements carried out in quiescent cardiomyocytes of Chagas-infected mice at different phases of infection. In cardiomyocytes from CONT mice [Na^+^]_d_ was 8 ± 0.2 mM; In cardiomyocytes from EAP-infected mice [Na^+^]_d_ was 9.4 ± 0.7 mM (*p*<0.001 compared to CONT cardiomyocytes); In cardiomyocytes from AP-infected mice [Na^+^]_d_ was 13.9 ± 0.8 mM (*p*<0.001 compared to CONT cardiomyocytes); and in cardiomyocytes from CP-infected mice [Na^+^]_d_ was 15.9 ± 1 mM (*p*<0.001 compared to CONT cardiomyocytes) (**Figure 2C**).

### Removal of [Na^+^]_e_ Increased [Ca^2+^]_d_ in Cardiomyocytes

**Figure 3:**
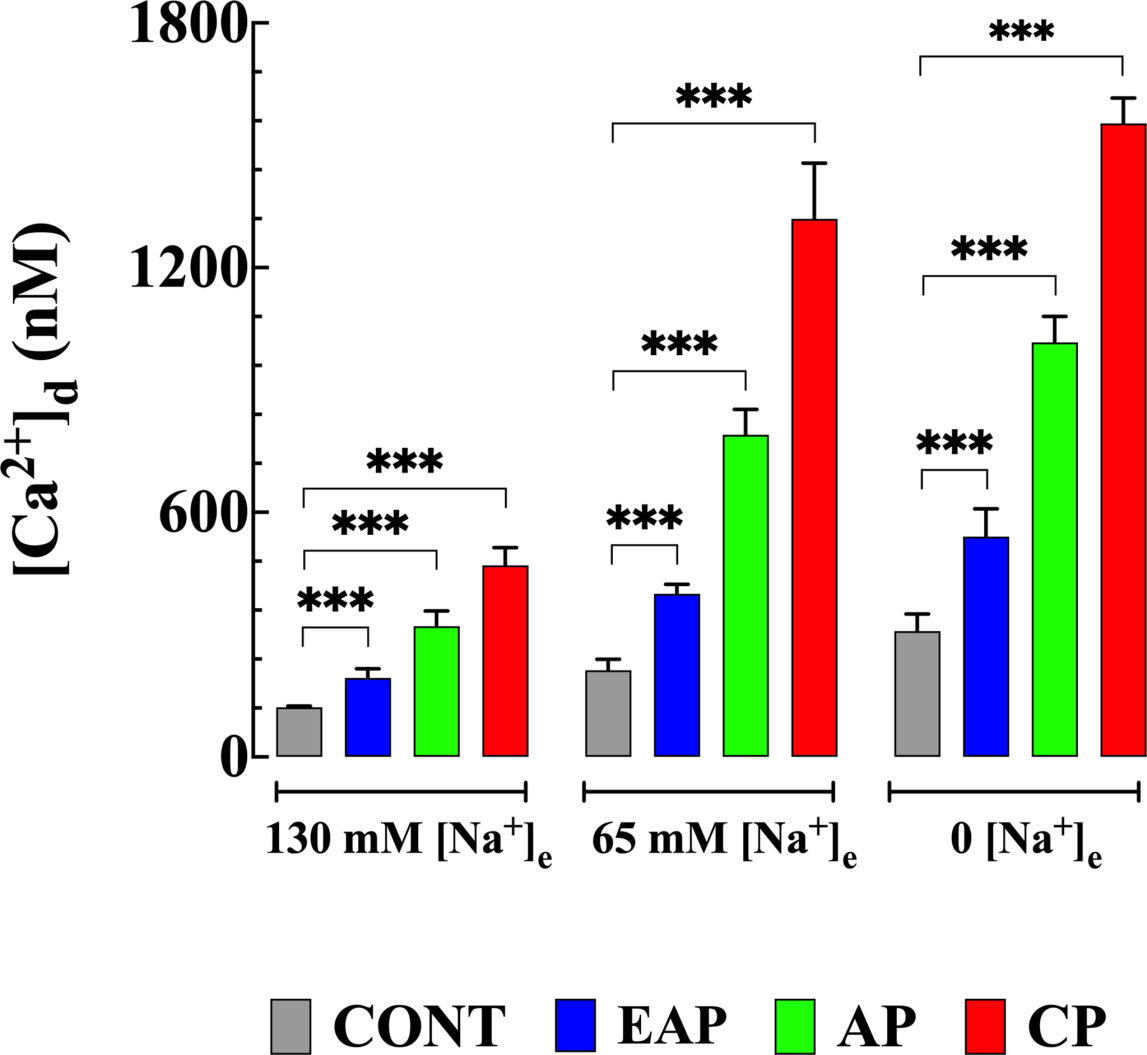
Effects of reduction in [Na^+^]_e_ on [Ca^2+^]_d_ in cardiomyocytes from control and Chagas infected mice. [Ca^2+^]_d_ was measured before and after cardiomyocyte treatments with solutions in which [Na^+^]_e_ was partially or completely replaced with NMG. [Ca^2+^]_d_ was determined in quiescent cardiomyocytes from CONT mice (*n_cells_* =13-24 and *n_mice_* =4), EAP-infected mice (*n_cells_* =12-19 and *n_mice_* =5), AP-infected mice (*n_cells_* =12-21 and *n_mice_* =5) and CP-infected mice (*n_cells_* =6-18 and *n_mice_* =9). Data are expressed as means ± S.D. Statistical analysis was performed as described above, ***p < 0.001. CONT, control mice; EAP, early acute phase of infected mice; AP, acute phase of infected mice; and CP, chronic phase of infected mice.

In a separate set of experiments, the reverse mode activity of NCX (Ca^2+^ entry in exchange for Na^+^ leaving the cell) was studied in quiescent cardiomyocytes isolated from control and infected mice by incubation in a solution in which Na^+^ was replaced in equivalent amounts by NMG. [Ca^2+^]_d_ was measured before and after incubation with a low or Na^+^ free solution. The reduction of [Na^+^]_e_ to 65 mM caused an increase in [Ca^2+^]_d_ in CONT cardiomyocytes to 212 ± 27 nm, in cardiomyocytes from EAP-infected mice to 400 ± 24 nM, in cardiomyocytes from AP infected mice to 790 ± 62 nM, and cardiomyocytes from CP-infected mice to 1,320 ± 137 nM (*p*<0.001 compared to cardiomyocytes from CONT, EAP, AP and CP-infected mice bathed in normal [Na^+^]_e_) (**Figure 3**).

Incubation in Na^+^ free solution caused a further increase in [Ca^2+^]_d_ in control and cardiomyocytes from infected mice. In CONT cardiomyocytes [Ca^2+^]_d_ increased significantly to 309 ± 42 nM, in cardiomyocytes from EAP-infected mice to 540 ± 69 nM, from AP-infected mice to 1,016 ± 64 nM, and from CP-infected mice to 1,523 ± 62 nM respectively (*p*<0.001 compared to cardiomyocytes from CONT, and EAP-, AP- and CP- infected mice bathed in normal [Na^+^]_e_). (**Figure 3**). The sustained increase [Ca^2+^]_d_ was maintained as long as Na^+^ was absent from the bath solution and reversed when Na^+^ was reintroduced into the Tyrode solution, except for cardiomyocytes from CP- infected mice where an irreversible elevation [Ca^2+^]_d_ associated with contracture was observed.

### Removal of Extracellular Ca^2+^ Prevents the Increase in [Ca^2+^]_d_ Induced by Na^+^ Depletion

**Figure 4:**
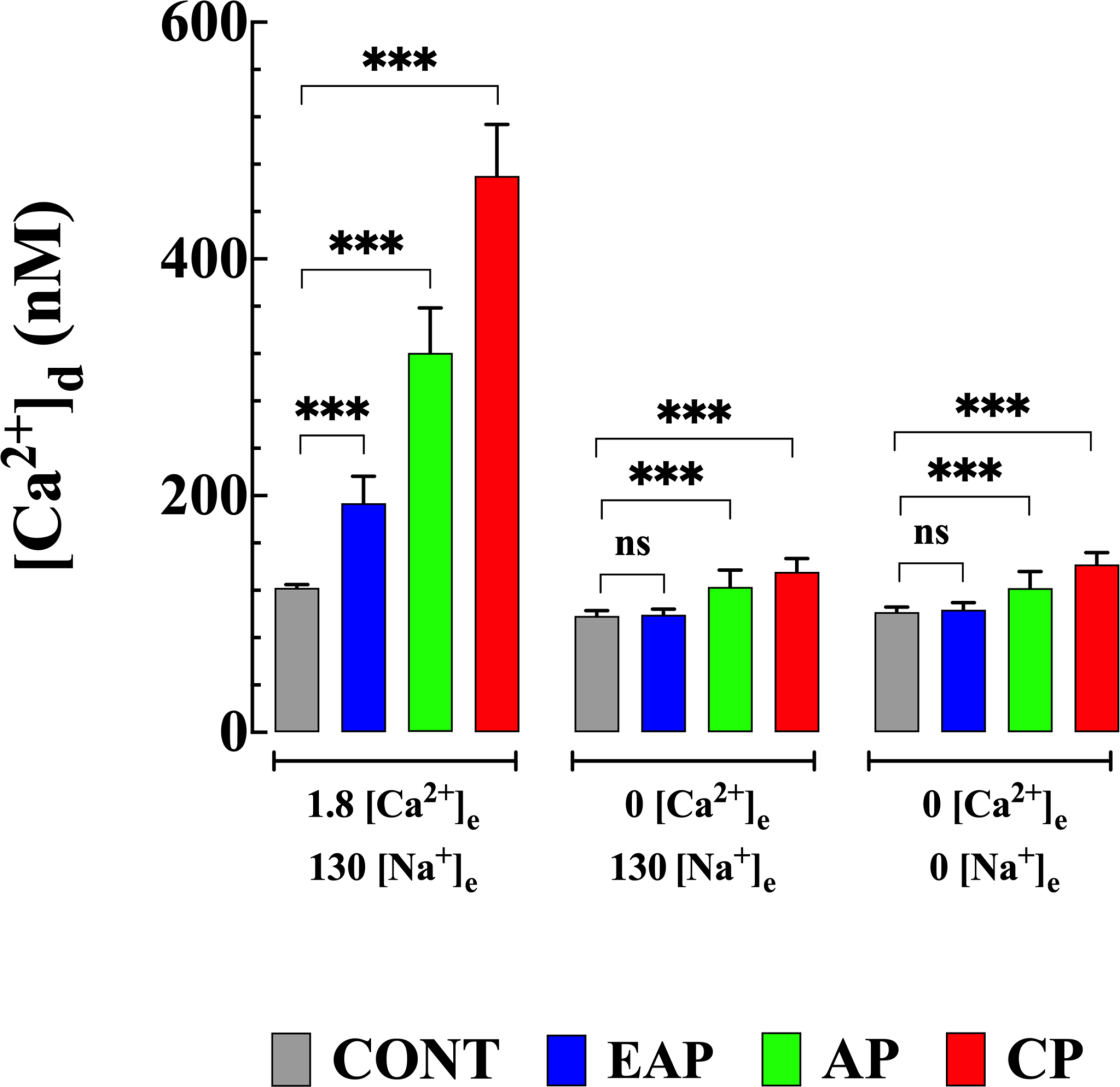
Effect extracellular Ca^2+^ on Na^+^ depletion-induced elevation of [Ca^2+^]_d_. [Ca^2+^]_d_ was measured in cardiomyocytes from CONT, EAP-, AP-, and CP-infected mice in normal Tyrode, Ca^2+^-free Tyrode solution, and Ca^2+^ and Na^+^ free Tyrode solution. [Ca^2+^]_d_ was determined in quiescent cardiomyocytes from CONT (*n_cells_* =16-24 and *n_mice_* =4), EAP-infect mice (*n_cells_* =15-19 and *n_mice_* =5), AP-infect mice (*n_cells_* =18-21 and *n_mice_* =6) and CP-infect mice (*n_cells_* =15-18 and *n_mice_* =8). Data are expressed as mean ± S.E. Statistical analysis was performed as described above, *** *p* < 0.001. CONT, control mice; EAP, early acute phase of infected mice; AP, acute phase of infected mice; and CP, chronic phase of infected mice. ns, no significant.

In a different set of experiments, we explored the contribution of the Ca^2+^ influx to the elevation of [Ca^2+^]_d_ elicited by the withdrawal of Na^+^ in quiescent cardiomyocytes from control and infected mice. Cardiomyocytes were incubated for 10 minutes in a Ca^2+^-free solution prior to the withdrawal of Na^+^ (see solutions). Incubation in a Ca^2+^-free solution significantly reduced [Ca^2+^]_d_ to 98 ± 5 nM in cardiomyocytes from CONT mice (*p*<0.001 compared to CONT cardiomyocytes in normal [Ca^2+^]_e_), to 100 ± 11 nM in cardiomyocytes from EAP-infected mice (*p*<0.001 compared to EAP cardiomyocytes in normal [Ca^2+^]_e_), to 123 ± 14 nM cardiomyocytes from AP-infected mice (*p*<0.001 compared to AP cardiomyocytes in normal [Ca^2+^]_e_), and to 136 ± 11 nM in cardiomyocytes from CP-infected mice (*p*<0.001 compared to CP cardiomyocytes in normal [Ca^2+^]_e_). (**Figure 4**). Elevation of [Ca^2+^]_d_ induced by withdrawal of Na^+^ was abolished in the absence of extracellular Ca^2+^ in all cardiomyocyte groups (**Figure 4**).

### Equilibrium Potentials

Using the Nernst equation and the actual values [Ca^2+^]_d_ and [Na^+^]_d_ found in the present study, the estimated *E*_Ca_ in cardiomyocytes from CONT mice was +133 mV, in cardiomyocytes from EAP-infected mice was +127 mV, in cardiomyocytes from AP-infected mice was +119 mV, and in cardiomyocytes from CP-infected mice were +116 mV. While the estimated *E*_Na_ in cardiomyocytes isolated from CONT mice was +74 mV, EAP-infected mice was +70 mV, AP-infected mice was +59 mV, and CP-infected mice was +56 mV. The equilibrium potential of the Na^+^/Ca^2+^ exchanger (*E*_NCX_) was -44 mV in cardiomyocytes from CONT, -46 mV from EA-infected mice, -61 mV from AP-infected mice, and –64 mV from CP-infected mice, respectively.

### KB-R7943 and YM-244769 Reduced [Ca^2+^]_d_ and Prevented the Elevation of Ca^2+^ Induced by the Na^+^ Free Solution

**Figure 5:**
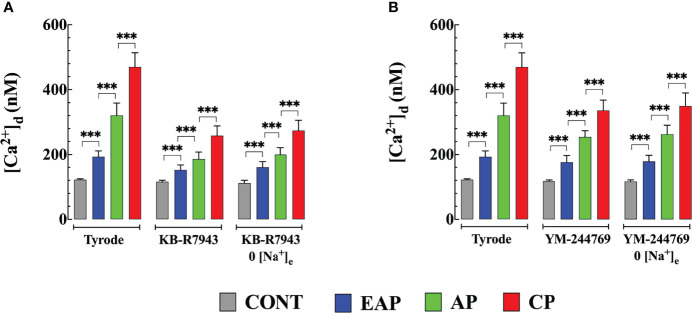
NCX blockers inhibited Na^+^ withdraw-induced [Ca^2+^]_d_ increases in cardiomyocytes from control and Chagas infected mice. **(A)** Effects of KB-R7943 and **(B)** YM-244769 on [Ca^2+^]_d_ increases caused by Na^+^ removal. [Ca^2+^]_d_ was determined in (i) normal Tyrode solution (ii) after 10 min of incubation with 10 μm KB-R7943) or 1 µM YM-244769 and (iii) after incubation, either KB-R7943 in Na^+^ free solution or YM-244769 in Na^+^ free solution. Experiments were carried out in cardiomyocytes from CONT (n_cells_=15-24 and n_mice_=6), EAP (n_cells_=12-19 and n_mice_=8), AP (n_cells_=13-21 and n_mice_=8) and CP cardiomyocytes (n_cells_=11-18 and n_mice_=9). Data are expressed as mean ± S.E. Statistical analysis was performed as described above, ***p < 0.001. CONT: control mice; EAP: early acute phase of infected mice; AP: acute phase of infected mice; and CP: chronic phase of infected mice.

To determine the mechanisms involved in the elevation of [Ca^2+^]_d_ caused by exposure to Na^+^ free medium, quiescent cardiomyocytes from control and infected mice were incubated in KB-R7943, a nonspecific blocker of the NCX reverse mode, which modified Na^+^ dependent binding (Iwamoto et al., 1996; Akabas, 2004). Cardiomyocyte preincubation with 10 μm KB-R7943 for 10 minutes significantly reduced [Ca^2+^]_d_ and inhibited the increase in [Ca^2+^]_d_ induced by the solution without Na^+^ (**Figure 5A**). KB-R7943 reduced [Ca^2+^]_d_ to 152 ± 16 nM in cardiomyocytes from EAP-infected mice, to 186 ± 21 nM from AP-infected mice, and 258 ± 30 nM from CP-infected mice (**Figure 5A**). No significant effect was observed in cardiomyocytes from CONT mice (116 ± 5 nM*).* Preincubation with KB-R7943 prevented the elevation of [Ca^2+^]_d_ caused by the Na^+^ free solution in cardiomyocytes of control and infected mice (compare **Figures 3**,**5A**).

Due to the lack of specificity of KB-R7943 (Abramochkin and Vornanen, 2014), we also examined the effects of YM-244769, a high-affinity blocker of the NCX reverse mode (Iwamoto and Kita, 2006). Incubation with 1 μm YM-244769 for 10 minutes reduced [Ca^2+^]_d_ to 176 ± 20 nM in cardiomyocytes from EAP-infected mice, to 254 ± 19 nM from AP-infected mice, and to 336 ± 32 nM from CP-infected mice. YM-244769 did not significantly reduce [Ca^2+^]_d_ in cardiomyocytes of CONT and EAP-infected mice. YM-244769 fully inhibited the elevation of [Ca^2+^]_d_ induced by the Na^+^ free solution in CONT cardiomyocytes and infected mice (compare **Figures 3**, **5B**).

## Discussion

Cardiac alterations are a severe and recurrent complication related to Chagas disease and represent the most common causes of heart failure and sudden death in Latin America (Velasco and Morillo, 2020). The present study reinforces our previous finding that progressive deterioration of cardiac function in Chagas cardiomyopathy is associated with defective intracellular Ca^2+^ regulation. In this report, we demonstrate, for the first time, that ventricular cardiomyocytes from rodents infected with *T. cruzi* show a partial depolarization associated with aberrant [Ca^2+^]_d_ and [Na^+^]_d_, which aggravated with endpoint after infection (chronic>acute>early acute). Furthermore, we provide evidence that chagasic infection enhances the reverse mode of NCX, causing an elevation of [Ca^2+^]_d,_ which is not mediated by membrane depolarization.

Calcium is essential in the regulation of cardiac contractility, and defective intracellular Ca^2+^ homeostasis plays an vital role in the pathogenesis of diverse cardiac diseases (Houser and Margulies, 2003; Lopez et al., 2011; Uryash et al., 2021a; Uryash et al., 2021b). [Ca^2+^]_d_ is regulated by several mechanisms that controls Ca^2+^ influx-efflux and intracellular reuptake that allow the maintenance of [Ca^2+^] within a physiological range (~100 nM) (Lopez et al., 2011; Mijares et al., 2014; Mijares et al., 2020). The [Ca^2+^]_d_ values obtained from CONT cardiomyocytes are in agreement with previous estimates obtained from humans (Lopez et al., 2011; Mijares et al., 2020) and non-human ventricular myocytes (Mijares et al., 2014; Uryash et al., 2021a; Uryash et al., 2021b) using Ca^2+^-selective microelectrodes and fluorescent Ca^2+^ indicator (Piacentino et al., 2003). The abnormal [Ca^2+^]_d_ in cardiomyocytes from infected mice (chronic>acute>early acute) agree with a similar dysfunction observed in cardiomyocytes obtained from Chagas patients and could potentially promote arrhythmias, similar to those observed in patients with Chagas cardiomyopathy (Benziger et al., 2017; Almeida et al., 2018).

Normal resting [Na^+^]_d_ in cardiomyocytes is in the range of 7-8 mM (Gray et al., 2001; Mijares et al., 2014), while in cardiomyocytes from infected mice, it was higher (CP>EP>AEP). Intracellular [Na^+^] is mainly regulated in cardiac myocytes by Na^+^/K^+^-ATPase, Na^+^/Ca^2+^ exchange, Na^+^ channels, and amiloride-sensitive Na^+^/H^+^ exchanger (Everts et al., 1992; Blaustein and Lederer, 1999; Bers et al., 2003). An elevated [Na^+^]_d_, as found in Chagas-infected mice cardiomyocytes, could change the balance of fluxes through NCX to favor a greater inflow of Ca^2+^ (reverse mode) (Blaustein and Lederer, 1999), contributing to intracellular Ca^2+^ overload as observed in Chagas-infected mice cardiomyocytes. Furthermore, it is plausible that the partial depolarization observed in cardiomyocytes from infected mice (chronic>acute>early acute) may be related to diastolic Na^+^ overload. Unfortunately, we did not explore whether the elevation of [Na^+^]_d_ in cardiomyocytes from infected mice was related to an increased influx or decreased efflux of Na^+^; therefore, no conclusions can be drawn.

Sodium-calcium exchange is the major Ca^2+^ efflux mechanism of ventricular cardiomyocytes. NCX mediates an electrogenic exchange of 3 Na^+^ for 1 Ca^2+^, which can occur in the forward (Ca^2+^ extrusion–Na^+^ entry) or in the reverse (Ca^2+^ entry– Na^+^ extrusion) mode (Blaustein and Lederer, 1999; Bers, 2000; Bers, 2002). In this study, we provide evidence for the first time of an enhancement in the reverse mode function of NCX in cardiomyocytes from infected mice (chronic>acute>early acute). The increase in [Ca^2+^]_d_ mediated by NCX was not associated with membrane depolarization and was dependent on extracellular [Ca^2+^] and [Na^+^] in all cardiomyocytes. Therefore, changes in NCX could contribute to intracellular Ca^2+^ overload, but could also contribute to the arrhythmogenesis observed in Chagas patients (Stein et al. 2018). The elevation of [Ca^2+^]_d_ was prevented in cardiomyocytes from control and infected mice by removing extracellular Ca^2+^ and reversed when Na^+^ was reintroduced into the bath, except for cardiomyocytes from CP-infected mice, where the removal of Na^+^ (partially or completely) produced irreversible contraction and cell death (40% in 65 mM [Na^+^]e and 82% in Na^+^ free solution, respectively).

The mode in which NCX operates is dependent on the Na^+^ and Ca^2+^ gradients across the sarcolemma, as well as the membrane potential, following a driving force equal to *E*_m_ – *E*_Na/Ca_ (where *E*_Na/Ca_= 3E_Na_ –2 *E*_Ca_ with *E*_Na_ and *E*_Ca_ represent the equilibrium potentials of Na^+^ and Ca^2+^, respectively) (Bers and Ginsburg, 2007). If the driving force for 3Na^+^ is greater than 1Ca^2+^ in the cell (3:1 Na^+^/Ca^2+^ stoichiometry), the NCX will transport Na^+^ into the cell and take Ca^2+^ out (forward mode). On the other hand, if under another set of conditions (altered membrane potential, ionic gradients, or post-translational modifications), the driving force for 3Na^+^ is less than that for 1Ca^2+^ in the cell, then the electrochemical gradient of Ca^2+^ will become the dominant inward driving force (reverse mode) (Bers and Ginsburg, 2007). The intracellular ionic changes in cardiomyocytes from infected mice modified the driving force (*E*_m_ − *E*_Na/Ca_) by 5 mV in cardiomyocytes from EAP-infected mice, 22 mV in cardiomyocytes from AP-infected mice, and 29 mV in cardiomyocytes from CP-infected mice.

KB-R7943 significantly reduced [Ca^2+^]_d_ by 1.3 times in cardiomyocytes from EAP-infected mice, 1.7 times in AP-infected mice, and 1.8 times in CP-infected mice, suggesting that the elevation of [Ca^2+^]_d_ observed in cardiomyocytes of Chagas infected mice was due in part to an influx of Ca^2+^ mediated by NCX in its reverse mode. On the contrary, in cardiomyocytes from CONT mice, KB-R7943 did not modify [Ca^2+^]_d_, indicating that in healthy quiescent cardiomyocytes, NCX in its reverse mode does not contribute significantly to [Ca^2+^]_d_. Furthermore, KB-R7943 prevented the elevation of [Ca^2+^]_d_ induced by Na^+^ withdrawal in cardiomyocytes from CONT and cardiomyocytes from infected mice. The involvement of NCX in the pathophysiology of Chagas disease is consistent with the recent publication by Santos-Miranda et al. (Santos-Miranda et al., 2021), which found the contribution of NCX as a cellular arrhythmogenic substrate in isolated cardiomyocytes from infected mice with the Colombian strain of *T*. *cruzi*. However, the effect of KB-R7943 on [Ca^2+^]_d_ must be considered with caution due to the lack of selectivity, since the NCX blocker also affects voltage-gated Na^+^ and Ca^2+^ channels, inward rectifying K^+^ channels in cardiac cells (Watano et al., 1996).

YM-244769 is a potent and highly selective NCX blocker that up to 1 µM (dose used in the present study) preferentially inhibits the reverse mode of NCX, with no effect on the forward mode (Iwamoto and Kita, 2006). YM-244769 significantly reduced [Ca^2+^]_d_ by 1.3 times in cardiomyocytes from AP- and 1.4 times from CP-infected mice. YM-244769 did not significantly reduce [Ca^2+^]_d_ in cardiomyocytes from CONT and EAP-infected mice, suggesting that the contribution of NCX to [Ca^2+^]_d_ is negligible. The effect of YM-244769 on [Ca^2+^]_d_ cardiomyocytes was small compared to KB-R7943, which is consistent with the fact that YM-244769 is a more specific NCX blocker and that KB-R7943 can act on other Ca^2+^ entry pathways. Furthermore, YM-244769 blocked the observed elevation of [Ca^2+^]_d_ caused by the Na^+^ free solution in all cardiomyocytes. Together, these data strongly support the hypothesis that Ca^2+^ entry mediated by the reverse NCX mode plays a significant role in [Ca^2+^]_d_ dyshomeostasis observed in cardiomyocytes isolated from Chagas infected mice during acute and chronic stages of infection. The fact that KB-R7943 or YM-244769 did not normalize [Ca^2+^]_d_ in cardiomyocytes from infected mice suggests the existence of additional Ca^2+^ pathways that appear to be altered due to Chagas infection.

### Conclusions

These results provide, for the first time, evidence that NCX plays an important role in aberrant diastolic [Ca^2+^] observed in cardiomyocytes from infected mice (chronic>acute>early acute). These novel findings may open a new therapeutic approach to improve cardiac function in patients suffering from chronic Chagas cardiomyopathy because there is still no effective treatment. Unfortunately, Chagas cardiomyopathy remains largely ignored despite its medical and social relevance.

### Study Limitations

Despite the novelty of this study, some limitations should be pointed out. We did not determine whether the elevation of [Na^+^]_d_ in cardiomyocytes from infected mice could be related to an increased influx or decreased efflux of Na^+^. Furthermore, the expression of NCX and other proteins involved in intracellular Ca^2+^ regulation were not studied.

## Data Availability Statement

The original contributions presented in the study are included in the article/supplementary material. Further inquiries can be directed to the corresponding author.

## Ethics Statement

The animal study was reviewed and approved by Care and Use Handbook of Laboratory Animals published by the US National Institute of Health (NIH publication No. 85-23, revised 1996) and approved by the Institutional Animal Care (IACUC) and Use Committees.

## Author Contributions

JRL: Designed research, performed the experiments, and analyzed the data; NL: Performed the experiments; JAA: Designed research; AM: Designed research, and analyzed the data. All authors contributed to manuscript revision, read, and approved the submitted version.

## Funding

This work was supported by the Florida Heart Research Foundation.

## Conflict of Interest

The authors declare that the research was conducted in the absence of any commercial or financial relationships that could be construed as a potential conflict of interest.

## Publisher’s Note

All claims expressed in this article are solely those of the authors and do not necessarily represent those of their affiliated organizations, or those of the publisher, the editors and the reviewers. Any product that may be evaluated in this article, or claim that may be made by its manufacturer, is not guaranteed or endorsed by the publisher.
